# Unrecognized Synergistic Effect of Spinal Epidural Lipomatosis on Spinal Stenosis and Its Radiological Grading: A Case Series

**DOI:** 10.7759/cureus.87439

**Published:** 2025-07-07

**Authors:** Ming Ho Tong, Hei Man Cheng, Kwun Man Mo, Chi Yeung Chu

**Affiliations:** 1 Department of Radiology, Pamela Youde Nethersole Eastern Hospital, Hong Kong, HKG

**Keywords:** borré classification, degenerative lumbar spine, epidural fat, metabolic syndrome, spinal canal stenosis, spinal epidural lipomatosis

## Abstract

Spinal epidural lipomatosis (SEL) is characterized by an excessive accumulation of epidural fat. It often exacerbates spinal stenosis and dural compression when combined with degenerative changes, leading to low back pain, radiculopathy, or myelopathy. Despite its increasing prevalence given rising obese population, the contribution of excessive epidural fat to spinal stenosis remains underrecognized on magnetic resonance imaging (MRI). This case series included four patients (aged 25-72 years) with varying degrees of SEL, lumbar spondylosis, and spinal stenosis. The clinical presentations were discussed. The amount of epidural fat on their lumbosacral spine MRI were graded by Borré classification (based on dural sac to fat ratio and epidural fat percentage), and the dural sac deformities were shown. We aim to improve radiologists' awareness on routine evaluation of epidural fat and its synergistic effect on spinal stenosis when combined with lumbar spondylosis to achieve early diagnosis. This serves to improve patient’s outcome as SEL can be reversed by conservative treatment, and its association with metabolic syndrome further potentiate timely risk factor control to avoid cardiovascular disorders.

## Introduction

Spinal epidural lipomatosis (SEL) is characterized by the accumulation of excessive unencapsulated fat in the epidural space, which may result in spinal stenosis, as well as compression on the dural sac and nerve roots [1]. It is not uncommon in clinical practice but is often underestimated for its significance, especially when there are co-existing spinal degenerative elements. Depending on the level of involvement, their synergistic effect on spinal stenosis may result in chronic low back pain, radicular leg pain, or, in severe cases, can come up with myelopathy [1-3]. While historically tied to chronic steroid use, obesity has emerged as the predominant cause in the context of a growing obese population [4]. Early diagnosis has great clinical impacts, as SEL is potentially reversible through weight loss or steroid cessation, offering symptom relief without surgery [5]. The detection and emphasis of underlying obesity may also motivate both the patient and clinician for weight loss, potentiating the prevention of metabolic syndrome. Magnetic resonance imaging (MRI) is the best modality to evaluate the extent of increased epidural fat, spinal degenerative changes, sign of mass effect on the dural sac and nerve roots. Additionally, it allows radiologist to evaluate the visceral and subcutaneous fat of patients. By presenting MRI images of patients with mild to severe SEL graded via the Borré classification, as well as the co-existing spinal degenerative changes, this case series aims to raise radiologists’ and clinicians’ awareness of the significance in exacerbating spinal stenosis. It further encourages the routine evaluation of epidural fat to achieve timely diagnosis and risk factor management.

## Case presentation

SEL grading system: Borré classification

The small amount of epidural fat in a normal individual usually ranges from 3 to 6 mm in thickness on sagittal plane [6]. The Borré classification was first described in 2003 for the objective grading of excessive epidural fat in patients with lumbar SEL [7]. The authors suggest performing the grading on an axial plane that is parallel and tangent to the superior endplate of the S1 vertebral body. Three measurements are utilized at the midline: the anteroposterior (AP) length of the spinal canal, the dural sac, and the epidural fat. Calculation of the ratio of dural sac to epidural fat (dura/fat ratio) and the percentage of epidural fat to spinal canal (fat percentage of spinal canal) are then performed.

Despite L5/S1 level is commonly involved in SEL, application of grading only to this single level may not accurately reflect the extent of excessive epidural fat in the rest of the lumbar spine. This is because conditions such as commonly seen L5/S1 spondylolisthesis and spinal operation can alter the amount of epidural fat. More recent article has suggested that the same measurement and ratios can be performed at other spinal levels, as well as on sagittal images, for SEL grading [2]. Table 1 summarizes the grading suggested by Borré et al.

**Table 1:** Borré classification for MRI grading of lumbar SEL. MRI: magnetic resonance imaging; SEL: spinal epidural lipomatosis. Source: Borré et al. [7].

Grade	Meaning	Fat% of spinal canal	Dura/fat ratio
Normal	Normal amount of epidural fat	≤40%	≥1.5
Grade I	Mild overgrowth of epidural fat	41-50%	1.49-1
Grade II	Moderate overgrowth of epidural fat	51-74%	0.99-0.34
Grade III	Severe overgrowth of epidural fat	≥75%	≤0.33

**Figure 1:**
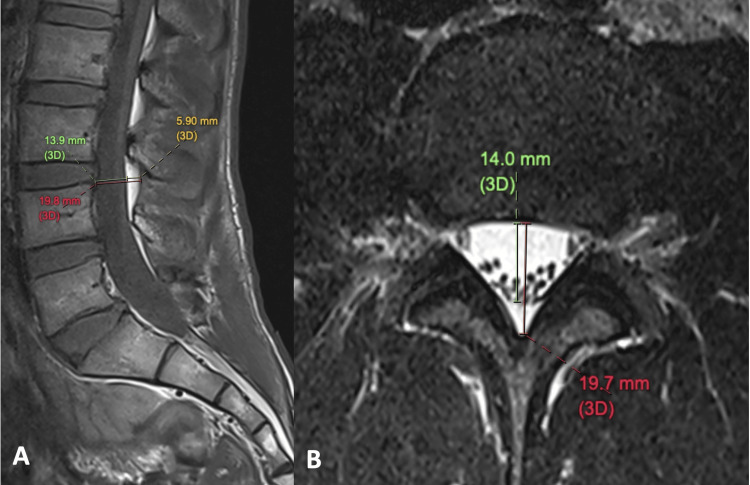
(A) Sagittal T1-weighted and (B) axial T2-weighted MRI of lumbosacral spine on a 30-year-old man with chronic low back pain. Red line: AP length of spinal canal; green line: AP length of dural sac; yellow line: AP length of epidural fat. MRI: magnetic resonance imaging.

For illustrative purposes, we applied the grading system on a young male patient with chronic low back pain (Figure 1). The anteroposterior (AP) length of the spinal canal (from vertebral body anteriorly to ligamentum flavum posteriorly) measures 19.7 mm. The dural sac measures 14.0 mm. The thickness of epidural fat (including fat anterior and posterior to dural sac) measures 5.7 mm. The measurements on the sagittal plane yields largely similar values compared with the axial plane. Thus, we obtained the following values for calculation: Dura/fat ratio = 14.0/5.7 = 2.5 and fat percentage of spinal canal = 5.7/19.7 x 100% = 28.9%. Both values are in line with normal grade of epidural fat.

Case 1

**Figure 2:**
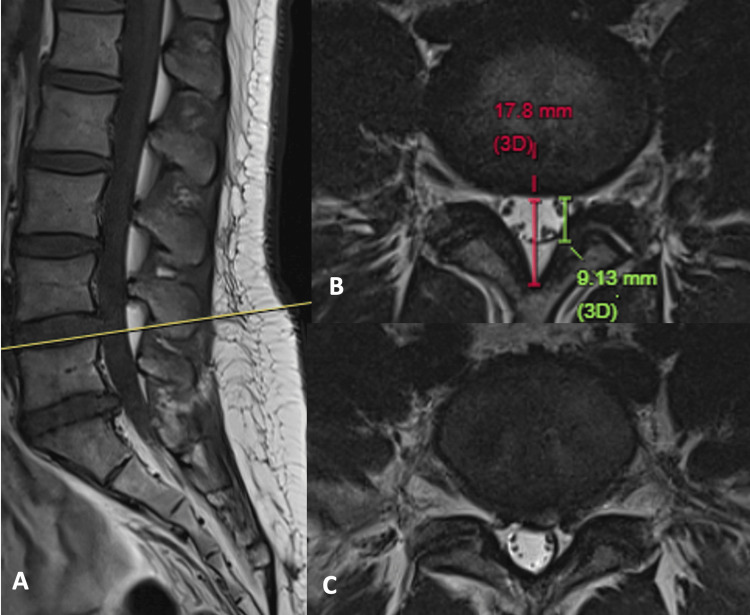
MRI of the lumbosacral spine of a 25-year-old woman with buttock pain radiating to the left leg. (A) Sagittal T1-weighted image shows prominent epidural fat, especially from L2 to S2 levels. (B) Axial T2-weighted image at L5 superior endplate shows a normal dural sac (dura/fat ratio = 1.05, fat% = 49%), mild SEL. (C) Axial T2-weighted image at the L5/S1 level shows posterior disc protrusion, causing bilateral lateral recess stenosis touching descending S1 nerve roots. This may explain patient’s radiculopathy. MRI: magnetic resonance imaging; SEL: spinal epidural lipomatosis.

A 25-year-old woman initially presented with buttock pain radiating to the left calf. She has no lower limb weakness or numbness. The straight leg raise test on the left side was positive at 70 degrees. Sagittal T1-weighted MRI of the lumbosacral spine shows thick subcutaneous fat, suggestive of underlying obesity. There is prominent epidural fat from L2 to S2 levels. L5/S1 disc protrusion with mild spinal stenosis is also seen (Figure 2A). An axial T2-weighted image at the L4/5 level, which has the greatest amount of epidural fat, shows no significant spinal stenosis. The normal oval configuration of the dural sac is preserved. The dura/fat ratio at this level is 1.05, and the percentage of epidural fat to the spinal canal is 49%, classified as mild SEL according to the Borré classification (Figure 2B). The axial T2-weighted image at the L5/S1 level better shows posterior disc protrusion with lateral recess stenosis touching bilateral descending S1 nerve roots (Figure 2C).

In this case, the likely cause of radiculopathy is L5/S1 disc protrusion, with co-existing SEL. On follow-up, the patient reported engaging in regular swimming exercise and achieving weight loss. The low back and left leg pain have subjectively reduced to ~70% of the baseline.

Case 2

**Figure 3:**
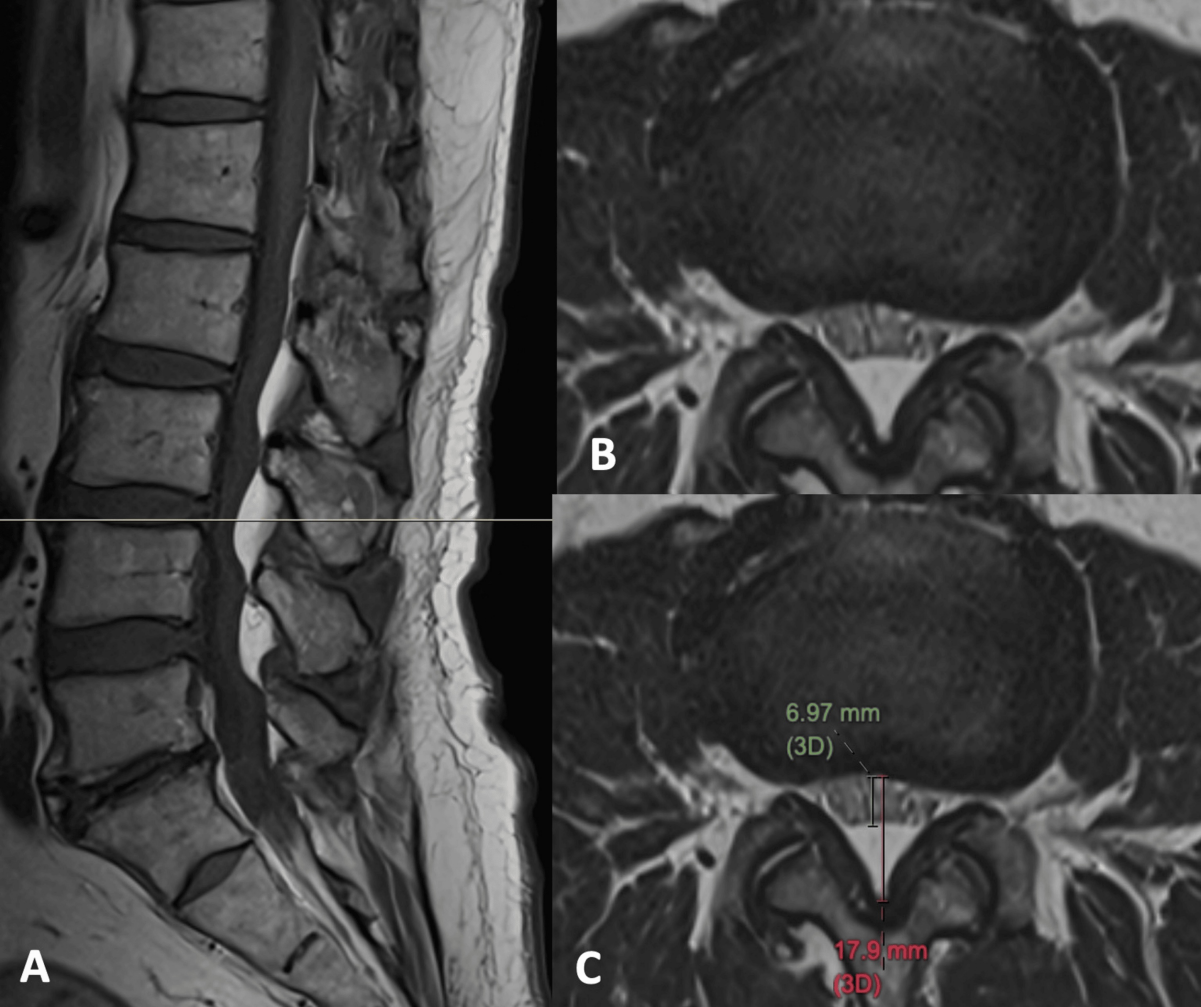
MRI of the lumbosacral spine of a 70-year-old man with a history of increasing low back pain for years with also radiation to the left leg. (A) Sagittal T1-weighted image shows increased epidural fat from the L2/3 to L4/5 levels. Lumbar spondylosis with marginal osteophytes and disc herniation are evident. (B, C) Axial T2-weighted images at the L3/4 level, near the superior endplate of L4, show an asymmetrical posterior disc herniation, which is more prominent on the left paracentral region. The dural sac is in polygonal shape with mild crowding of the cauda equina (dura/fat ratio = 0.64, fat% = 61%), moderate SEL. MRI: magnetic resonance imaging; SEL: spinal epidural lipomatosis.

A 70-year-old man with a history of obstructive sleep apnea and known obesity. He complained of increasing low back pain for years with radiation to the left leg. Upon physical examination, no lower limb weakness or numbness was noted. Sagittal T1-weighted MRI of the lumbosacral spine shows thick subcutaneous fat and increased epidural fat from the L2/3 to L4/5 levels. Lumbar spondylosis with marginal osteophytes and disc herniation are evident (Figure 3A). An axial T2-weighted image at the L3/4 level shows an asymmetrical posterior disc herniation, which is more prominent at the left paracentral region. The synergistic effect of increased epidural fat and disc herniation results in moderate spinal stenosis. The dural sac is in polygonal shape with mild crowding of the cauda equina. The dura/fat ratio is 0.64, and the percentage of epidural fat to spinal canal is 61%, classified as moderate SEL (Figure 3B, C).

Case 3

A 60-year-old man, who underwent clipping for an anterior communicating artery aneurysm, complained of progressive numbness and weakness in bilateral lower limb over the past three months. He also has a known history of chronic low back pain for more than 10 years. Recently, he can only walk for 15 minutes due to worsening symptoms. Physical examination showed reduced power and sensation at the bilateral L2 and L3 levels.

**Figure 4:**
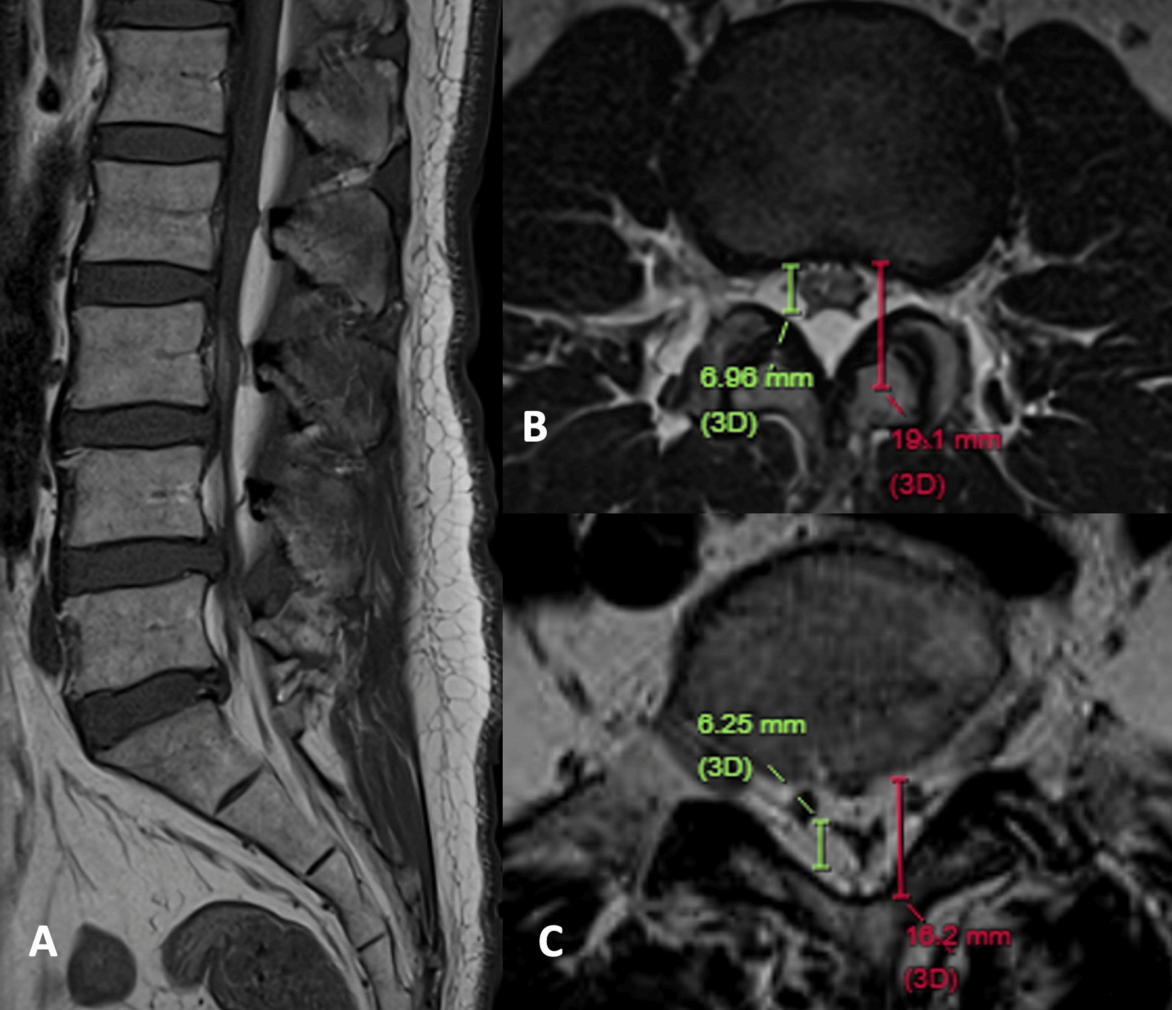
MRI of the lumbosacral spine of a 60-year-old man with chronic lower back pain and increasing spinal claudication. (A) Sagittal T1-weighted image shows a loss of lumbar lordosis and prominent epidural fat from L1 to S2 levels. (B) Axial T2-weighted image at L2/3 shows increased posterior epidural fat and degenerative changes, resulting in moderate spinal stenosis with polygonal dural sac (dura/fat ratio = 0.58, fat% = 63%). (C) Axial T2-weighted image at L5/S1 level revealed a Y-shaped dural sac from anterior and posterior epidural fat compression (dura/fat ratio= 0.63, fat% = 61%). Both levels are moderate SEL, with cauda equina crowding. MRI: magnetic resonance imaging; SEL: spinal epidural lipomatosis.

Sagittal T1-weighted MRI of the lumbosacral spine shows a loss of lumbar lordosis, thick subcutaneous fat, and increased epidural fat from L1 to S2 levels (Figure 4A). An axial T2-weighted image at the L2/3 level shows that the combined effects of posterior epidural fat, mild disc bulging, and facet arthrosis result in moderate spinal stenosis and polygonal configuration of dural sac. The dura/fat ratio is 0.58, and the percentage of epidural fat to spinal canal is 63% (Figure 4B). At the L5/S1 level, both anterior and posterior epidural fat compress the dural sac to “Y” shape. The dura/fat ratio is 0.63, and the percentage of epidural fat to spinal canal is 61% (Figure 4C). Both are moderate SEL. The synergistic effect of increased epidural fat and degenerative changes results in crowding of the cauda equina. The patient was referred to the orthopedic team and suggested lifestyle modification for losing weight.

Case 4

A 72-year-old man was referred to the orthopedic clinic for chronic low back pain, buttock pain, and progressive spinal claudication after walking for 30 minutes. He also reported numbness in both anterior shins. He has a known history of diabetes, hyperlipidemia, and hypertension. Physical examination showed reduced power and sensation at the L4 and L5 levels (Left>Right).

**Figure 5:**
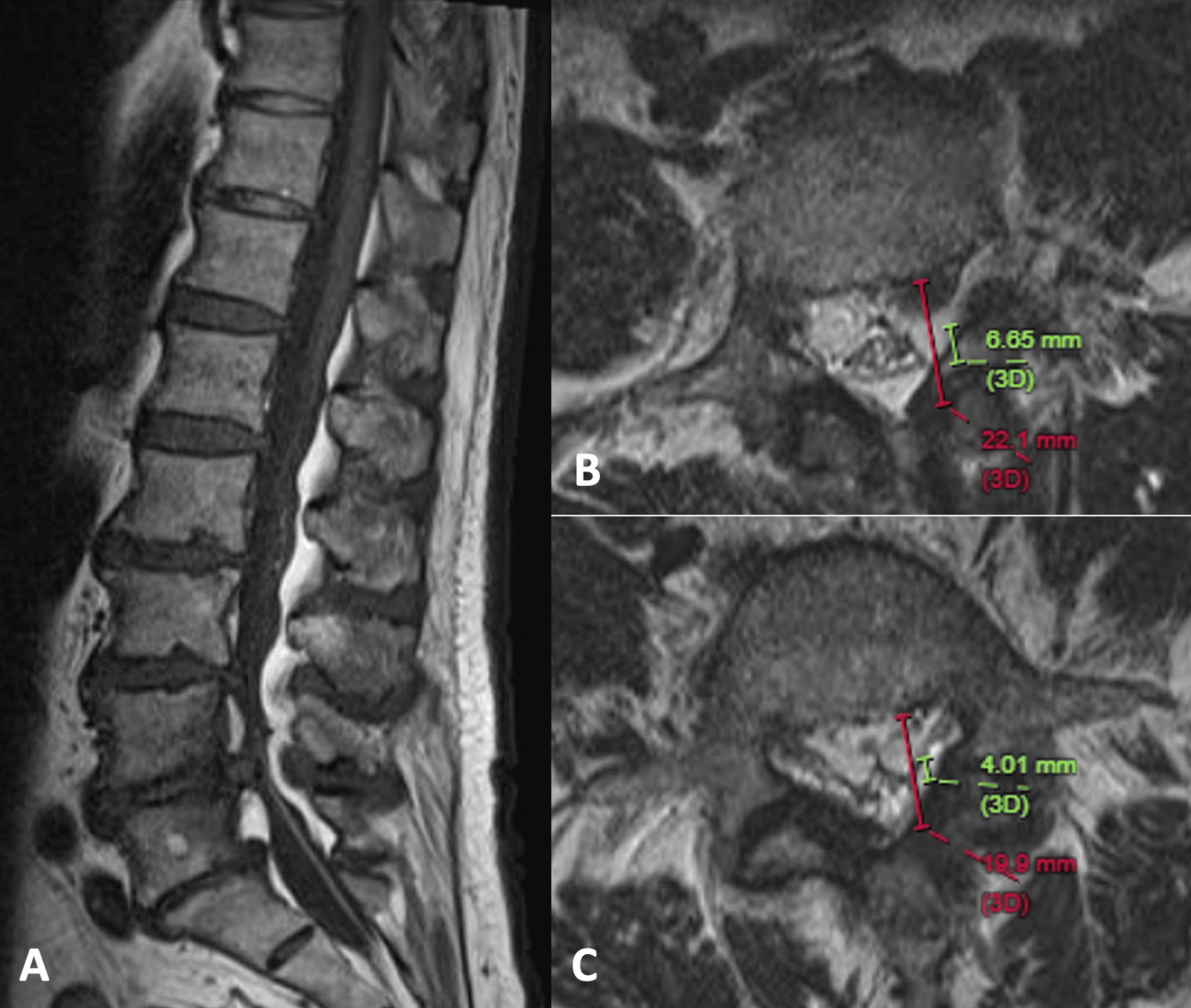
MRI of the lumbosacral spine of a 72-year-old man with chronic lower back pain and increasing spinal claudication. (A) Sagittal T1-weighted image shows excessive epidural fat, especially at the L3/4 to L5/S1 levels, both anterior and posterior to the dural sac. The combined effect of excessive epidural fat and degenerative changes results in severe spinal stenosis. (B) Axial T2-weighted image near the L3/4 level shows a polygonal dural sac (dural/fat ratio: 0.45, fat%: 70%), moderate SEL. (C) Axial T2-weighted image near the L4/5 level shows a stellate dural sac (dura/fat ratio: 0.25, fat%: 80%), severe SEL. MRI: magnetic resonance imaging; SEL: spinal epidural lipomatosis.

Sagittal T1-weighted MRI of the lumbosacral spine shows thick subcutaneous fat and excessive epidural fat, especially at L3/4 to L5/S1 levels, both anterior and posterior to the dural sac. Lumbar spondylosis with marginal osteophytes and disc herniation are seen. The combined effect of excessive epidural fat and degenerative elements results in severe spinal stenosis, compression onto the dural sac with redundant nerve roots, suggestive of chronic cauda equina compression (Figure 5A). Axial T2-weighted image near the L3/4 level shows a polygonal dural sac and measurements of moderate SEL (Figure 5B). Near the L4/5 level, there is a stellate configuration of the dural sac due to circumferential epidural fat compression. The dura/fat ratio is 0.25, and the percentage of epidural fat to spinal canal is 80%, classified as severe SEL (Figure 5C). The patient was offered decompression surgery, but he refused due to worry on surgical risk.

## Discussion

SEL, once considered a rare condition, is now increasingly encountered on spinal MRI in the context of a growing obese population. Obesity is now the leading cause, followed by exogenous steroid use [2]. Less common causes include idiopathic SEL and endocrinopathies like Cushing’s syndrome [1].

Our case series highlights the radiological features and grading system of SEL in patients with chronic low back pain, radiculopathy, and spinal claudication. Despite its distinct imaging appearance (epidural high T1 signal showing suppression on fat-saturated sequences and associated mass effect onto dura), SEL is often overlooked by radiologists. A recent retrospective analysis revealed that the reporting rate of SEL only accounts for 8% of the cases [8]. In clinical practice, when the spinal canal is narrowed only by a profound amount of epidural fat, it is easy to recognize the excessive fat as the culprit of patients’ symptoms. However, when there are concomitant degenerative changes like disc herniation or facet arthropathy, the focus to review the epidural fat can be lost. As illustrated by our cases, SEL is more likely to result in significant spinal stenosis and generate symptoms when there is co-existing spinal degeneration. Even if there are only mild degenerative changes, the additional mass effect from excessive epidural fat can be significant enough to result in dural sac compression and even cauda equina compression. Commonly noticed dural sac deformities are polygonal, stellate, and Y-configurations. Thus, radiologists should improve their awareness of any epidural fat overgrowth, as well as learn to grade its amount.

In the past, surgical removal of epidural fat was the primary treatment for SEL. However, the surgical success in improving patients’ subjective symptoms from spinal stenosis varies among different patients. Ferlic et al. conducted a retrospective study following patient outcomes after surgical treatment of lumbar SEL, showing around half of the patients reported clinically relevant improvement, and the difference remained significant only for up to two years [9]. Meanwhile, surgical decompression is not without risk, and obesity itself is known to be an important surgical risk factor [10]. Given the reversibility of epidural fat deposition, conservative management targeting the causative factors is now the preferred approach. Cessation of the offending exogenous steroid and weight reduction in obesity-induced SEL have been proven effective in reducing epidural fat and alleviating patients’ symptoms. Surgery is considered when conservative treatment fails or the symptoms are severe, such as patient presenting with cauda equina compression [1,5,11].

The health implications of early diagnosis of SEL do not stop with the spine but also potentiate early identification of metabolic syndrome, which is linked to increased cardiovascular risk and reduced life expectancy. This syndrome is closely associated with visceral fat deposition rather than subcutaneous fat deposition. Studies have proven that SEL is a hallmark of metabolic syndrome due to its statistically significant correlation with the amount of visceral fat, body mass index, and abdominal circumference [2]. Timely diagnosis of SEL may serve to flag up patients with possible metabolic syndrome for more aggressive treatment before devastating outcome occurs.

## Conclusions

SEL is an important condition to be recognized. Its synergistic effect with background lumbar spondylosis can exacerbate spinal stenosis, leading to dural or cauda equina compression, which produces chronic low back pain and radiculopathy in patients. Radiologists should be familiar with the grading system and imaging features, as early diagnosis can reproduce potential benefits of reversing the condition without surgery and identify patients at increased risk of metabolic syndrome.
